# Targeting IL-1β and IL-17A Driven Inflammation during Influenza-Induced Exacerbations of Chronic Lung Inflammation

**DOI:** 10.1371/journal.pone.0098440

**Published:** 2014-06-11

**Authors:** Anke Sichelstiel, Koshika Yadava, Aurélien Trompette, Olawale Salami, Yoichiro Iwakura, Laurent P. Nicod, Benjamin J. Marsland

**Affiliations:** 1 Faculty of Biology and Medicine, University of Lausanne, Service de Pneumologie, CHUV, Lausanne, Switzerland; 2 Molecular Biomedicine, ETHZ, Zurich, Switzerland; 3 Research Institute for Biomedical Sciences, Tokyo University of Science, Tokyo, Japan; Helmholtz Zentrum München/Ludwig-Maximilians-University Munich, Germany

## Abstract

For patients with chronic lung diseases, such as chronic obstructive pulmonary disease (COPD), exacerbations are life-threatening events causing acute respiratory distress that can even lead to hospitalization and death. Although a great deal of effort has been put into research of exacerbations and potential treatment options, the exact underlying mechanisms are yet to be deciphered and no therapy that effectively targets the excessive inflammation is available. In this study, we report that interleukin-1β (IL-1β) and interleukin-17A (IL-17A) are key mediators of neutrophilic inflammation in influenza-induced exacerbations of chronic lung inflammation. Using a mouse model of disease, our data shows a role for IL-1β in mediating lung dysfunction, and in driving neutrophilic inflammation during the whole phase of viral infection. We further report a role for IL-17A as a mediator of IL-1β induced neutrophilia at early time points during influenza-induced exacerbations. Blocking of IL-17A or IL-1 resulted in a significant abrogation of neutrophil recruitment to the airways in the initial phase of infection or at the peak of viral replication, respectively. Therefore, IL-17A and IL-1β are potential targets for therapeutic treatment of viral exacerbations of chronic lung inflammation

## Introduction

Chronic obstructive pulmonary disease (COPD) is currently ranked the 4^th^ leading cause of death worldwide by the World Health Organization (WHO), and its incidence is increasing. The main risk factor of COPD is exposure to tobacco smoke which triggers a cascade of inflammatory pathways leading to disease induction in susceptible people. Major hallmarks of the disease pathology are the development of emphysema and chronic bronchitis that lead to a progressive and irreversible airflow limitation resulting in a continuous decline of lung function [1]. COPD severity has been associated with acute periods of disease worsening [2], [3], so-called exacerbations, a key factor in COPD morbidity and mortality [4], [5]. By causing acute respiratory distress, they impact on the quality of patient’s health [6] and are responsible for most hospital stays related to the disease [4].

Exacerbations are primarily caused by respiratory viral or bacterial infections. Amongst those, viral-induced exacerbations account for approximately half of the cases [7], [8] and are associated with more severe acute episodes and prolonged recovery time [9]–[11]. The most common viral pathogen in exacerbated patients is rhinovirus, followed by influenza virus, RSV and coronavirus [7], [8], [10], [12]. Due to targeted vaccination of high risk groups, influenza infections occur less frequently in COPD patients of westernized countries [11]. However, they continue to be the predominant cause of viral exacerbations in Hong Kong [13] and Singapore [14].

COPD exacerbations have been linked to excessive inflammatory responses, including enhanced recruitment of inflammatory cells [15] and upregulation of a variety of proinflammatory mediators [16], [17]. Nevertheless, the underlying mechanisms and the most effective therapeutic strategies are still poorly understood and first-line therapy still predominantly relies on corticosteroids and bronchodilators [18], which are limited in their efficacy [17], [19]. Thus, the study of cellular and molecular mechanisms leading to exacerbations is key for the identification of urgently required therapeutic targets. One of the proinflammatory cytokines that has been associated with COPD is IL-1β, a major player in initiation and persistence of inflammation. In animal models mimicking features of COPD, IL-1 has been shown to be key to the induction of emphysema and inflammation [20]–[27]. Furthermore, its expression is significantly enhanced in COPD patients during acute episodes of exacerbations [17], [20], [28], [29]. Unraveling the role of IL-1β in viral exacerbations might therefore not only result in an overall better understanding of mechanisms of exacerbations, but also indicate whether it qualifies as a valid therapeutic target. A promising candidate for therapeutic inhibition of IL-1β signaling is one of its inhibitors, the interleukin-1 receptor antagonist (IL-1Ra) anakinra (Kineret, Amgen), which has been used effectively in treatment of rheumatoid arthritis.

In order to investigate the role of IL-1β during COPD exacerbations we utilized a model of LPS and elastase induced chronic lung inflammation, followed by infection with influenza in wild type or IL-1β deficient mice. We found that IL-1β was a key driver of pulmonary inflammation, primarily concerning recruitment of neutrophils and lung dysfunction. IL-1β driven neutrophilia was mediated by IL-17A in the initial phase of viral infection, but became independent of IL-17A during the peak phase of viral replication. Treatment with the IL-1Ra, anakinra, proved efficient in reducing neutrophilic inflammation at the peak of viral replication while blocking of IL-17A abrogated neutrophilia in the early phase of viral infection. Taken together our data indicate that blockade of IL-1β and IL-17A could be valid therapeutic approaches for treatment of virus-induced COPD exacerbations.

## Materials and Methods

### Ethics Statement

All animal experiments were performed according to institutional guidelines and Swiss federal and cantonal laws on animal protection. Animal experiments were approved by the following ethical committee: Service de la consommation et des affaires vétérinaires, Affaires vétérinaires, Canton de Vaud, Switzerland (permit numbers 2283 and 2216).

### Mice

C57BL/6 or BALB/c mice were between 8–12 weeks of age and were purchased from Charles River Laboratories. IL-1β deficient mice on C57BL/6 background were received from Prof. Iwakura [30], Tokyo University of Science, Japan, and bred in house.

### LPS/elastase Exposure and Viral Infection

**Figure 1:**
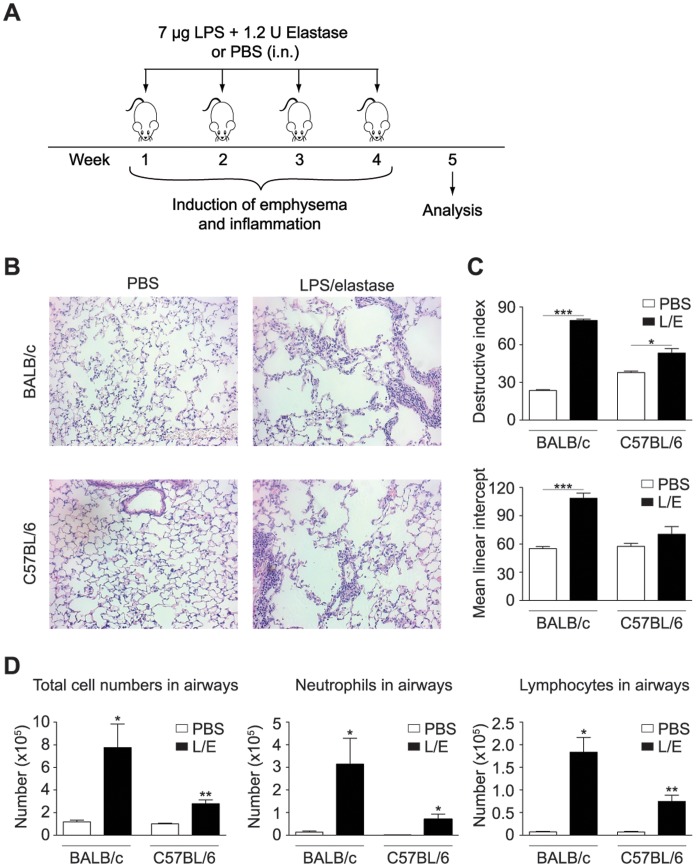
Model of chronic lung inflammation in BALB/c and C57BL/6 mice. BALB/c or C57BL/6 mice were exposed to LPS/elastase (L/E) or PBS once per week for four consecutive weeks as depicted in Figure 1A. Disease severity was determined one week after the last LPS/elastase challenge. (B) Histological sections of lungs were stained with hematoxylin and eosin (H&E). (C) Destructive index (DI) and mean linear intercept (Lm) were scored from histological sections. (D) Cellular influx into airways was assessed by differential cell counts. All data are representative of at least two independent experiments (n = 3–5), error bars indicate standard error of the mean (s.e.m).

Mice were exposed intranasally to a mixture of 7 µg LPS from *E. coli* O26:B6 (Sigma-Aldrich) and 1.2 U porcine pancreatic elastase (EPC) in a total volume of 100 µl, and treated once per week for four consecutive weeks (Figure 1A). Control mice were treated with PBS. Two weeks after the last LPS/elastase challenge, mice were infected with 250 PFU influenza A virus, strain PR8 (A/Puerto Rico8/34, H1N1, Viropur). The virus was administered intranasally in a total volume of 50 µl PBS; control mice received only PBS. For all intranasal administrations C57BL/6 mice were anesthetized by intraperitoneal injection of 54.17 mg/kg Ketamin (Ketasol-100, Graeub) and 1.28 mg/kg Xylazin (Xylasol, Graeub) and BALB/c mice with 78 mg/kg Ketamin and 1.93 mg/kg Xylazin.

### Histology and Quantification of Damage

Lungs were inflated with 1 ml 10% formalin, embedded into paraffin and stained with hematoxylin and eosin. Stained slides were analyzed by light microscopy. Pulmonary emphysema was quantified using Image J software by measuring the mean linear intercept for airspace enlargement and destruction index for alveolar wall destruction. 10 fields of view at 20X magnification per section of lung were used for quantification as described previously [31], [32].

### Quantification of Airway Inflammation

Airway cells were recovered by bronchoalveolar lavage and either analyzed by flow cytometry as described below or spun onto slides for differential cell counts. Slides were stained with Diff-Quik (Dade) and counts were performed according to standard criteria.

### Assessment of Pulmonary Resistance

Total lung resistance was measured using the whole body restrained plethysmograph system flexiVent from Scireq. Mice were anesthetized by intramuscular injection of 100 mg/kg ketamine (Ketasol-100, Graeub) and intraperitoneal injection of 50 mg/kg pentobarbital (Esconarkon, Streuli Pharma). Subsequently, mice were tracheotomized and mechanically ventilated at a rate of 450 breaths/min and a tidal volume of 10 ml/kg bodyweight.

### Flow Cytometry

Single cell suspensions from the whole lung including airways and trachea were obtained by digestion with 2 mg/ml Collagenase IV (Invitrogen) and 50 U/ml DNaseI (Roche). Neutrophils and monocytes in lung and bronchoalveolar lavage fluid were distinguished by staining with CD11c APC-Cy7, CD11b PerCP-Cy5.5, Ly-6G Biotin, Ly-6C Pacific Blue, Streptavidin PE-Cy7. Neutrophils were defined as CD11c^−^ CD11b^+^ Ly-6C^+^ Ly-6G^+^ and inflammatory monocytes as CD11c^−^ CD11b^+^ Ly-6C^+^ Ly-6G^low−intermediate^ as precised in detail in Figure S1A.

To analyze IL-17A production, cells from lung digests were stimulated with 10^−7^ M PMA, 1 µg/ml Ionomycin and 2×10^−6^ M Monensin for 4 h at 37°C (indicated chemicals were purchased from Sigma-Aldrich). Subsequently, cell subsets were distinguished by surface staining for CD4 PerCP-Cy5.5, CD8b FITC, γδ TCR Biotin, CD3 Pacific Blue, Streptavidin PE-Cy7 (Figure S1B) and IL-17A production was characterized by intracellularly staining with IL-17A Alexa700 after fixation with BD lysis buffer (BD Biosciences). All antibodies were purchased from Biolegend. Stained cells were acquired on a BD FACS CANTO or BD FACS LSRII and analyzed by using FlowJo software (Tree Star).

### Antibodies for *in vivo* Studies

For neutralization of IL-17A, mice were treated with 250 µg of anti-IL-17A (clone 17F3) or the corresponding isotype control antibody (clone MPOC-21) from BioXCell. The clone 17F3 uniquely reacts with the IL-17A and no other IL-17 isoform [33]. Antibodies were administered intraperitoneally one day before viral infection and two days post infection.

To block IL-1β signaling mice received 200 µg of the interleukin-1 receptor antagonist (IL-1Ra) anakinra (Kineret, Amgen) twice daily while control mice received only PBS. Anakinra was kindly provided by Prof. Alexander So (Centre Hospitalier Universitaire Vaudois, Lausanne, Switzerland) and Mme Ghislaine Aubel (Centre Hospitalier Universitaire Vaudois, Lausanne, Switzerland).

### Quantitative Real-time PCR

Total RNA was isolated from lung and trachea with TRI reagent (Molecular Research). All RNA samples were checked for purity using a NanoDrop 1000 spectrophotometer (Thermo Scientific) and met standard quality criteria. RNA was subsequently transcribed into cDNA by the iScript cDNA Synthesis kit (Bio-Rad) and quantitative real-time PCR was performed according to the manufacturer’s instructions utilizing the SsoAdvanced SYBR Green Supermix from Bio-Rad. Expression was determined by comparative delta-threshold cycle method using GAPDH as a comparator. The following primers were used: GAPDH forward 5′-GGG TGT GAA CCA CGA GAA AT-3′, GAPDH reverse 5′-CCT TCC ACA ATG CCA AAG TT-3′; CXCL1 forward 5′-GCC TAT CGC CAA TGA GCT G-3′, CXCL1 reverse 5′-ATT CTT GAG TGT GGC TAT GA-3′; CXCL2 forward 5′-AGT GAA CTG CGC TGT CAA TG-3′, CXCL2 reverse 5′-GCC CTT GAG AGT GGC TAT GAC-3′; CXCL5 forward 5′-AGC ATC TAG CTG AAG CTG CCC C-3′, CXCL5 reverse 5′-CCG TAG GGC ACT GTG GAC CTG-3′; G-CSF forward 5′-TGA CAC AGC TTG TAG GTG GC-3′, G-CSF reverse 5′-TCC TGC TTA AGT CCC TGG AG-3′; IL-6 forward 5′-TTC CAT CCA GTT GCC TTC TTG-3′, IL-6 reverse 5′-TCA TTT CCA CGA TTT CCC AGA G-3′; IL-17A forward 5′-ACC CTG GAC TCT CCA CCG CAA-3′, IL-17A reverse 5′-GGT GGT CCA GCT TTC CCT CCG-3′; influenza matrix protein forward 5′-GGA CTG CAG CGT AGA CGC TT-3′, influenza matrix protein reverse 5′-CAT CCT GTT GTA TAT GAG GCC CAT-3′; TNFα forward 5′-GCC AGG AGG GAG AAC AGA AAC-3′, TNFα reverse 5′-GCC AGT GAG TGA AAG GGA CAG-3′.

### ELISA

To determine cytokine levels whole lung and trachea were collected and stored in protease inhibitor solution (Roche) at −20°C until use. Tissue homogenate was prepared using a TissueLyser (Qiagen). IL-1β protein was determined using the mouse IL-1β ELISA kit Ready-SET-Go! from eBioscience by following the manufacturer’s instructions. IL-6 and TNFα were measured in a sandwich ELISA using 2.5 µg/ml anti-mouse IL-6 or TNFα (Biolegend) for coating. Bound protein was detected by 1 µg/ml biotin labeled anti-IL-6 or anti-TNFα antibody (Biolegend) and subsequent incubation with alkaline phosphatase conjugated streptavidin (Biolegend). Plates were developed using the substrate p-Nitrophenyl phosphate (Sigma-Aldrich) and OD was measured using an ELISA reader (Biotek).

### Statistical Analysis

Statistical significant differences were assessed using the Student’s *t* test (two tailed, unpaired). *P*-values below 0.05 were considered significant and were depicted with p≤0.05 (*), p≤0.005 (**), p≤0.0005 (***). Standard error of the mean was applied.

## Results

### Influenza-induced Exacerbations are Characterized by Neutrophilic Inflammation and Increased Airway Resistance

COPD is a heterogeneous disease in humans but core features of its pathology can be reproduced in mice by repetitive exposure to lipopolysaccharide (LPS) and elastase [34]. LPS is a bacterial endotoxin present in tobacco smoke [35], [36], the predominant risk factor of COPD. It has been shown to cause inflammation, and particularly when co-administered with elastase, chronic emphysema-like changes develop in mouse lungs [34]. As such, we exposed mice once a week for 4 consecutive weeks to a mixture of 7 µg LPS and 1.2 U porcine pancreatic elastase via the intranasal route, as depicted in Figure 1A. By this we induced strong emphysema (Figure 1B,C) and sustained pulmonary inflammation (Figure 1B,D) in BALB/c and C57BL/6 mice. The development of emphysema was scored by increases in the mean linear intercept and the destructive index (Figure 1C) from lung histology. In addition, we observed a strong airway inflammation, mainly driven by neutrophils and lymphocytes (Figure 1D). Both, emphysema and inflammation, remained above baseline levels for at least 2 months (data not shown). Thus, the response induced by repeated challenges with LPS/elastase resembled the pathology of COPD. As the disease severity was more pronounced in the BALB/c strain (Figure 1B–D), all experiments performed in wild type mice were conducted in BALB/c mice.

**Figure 2:**
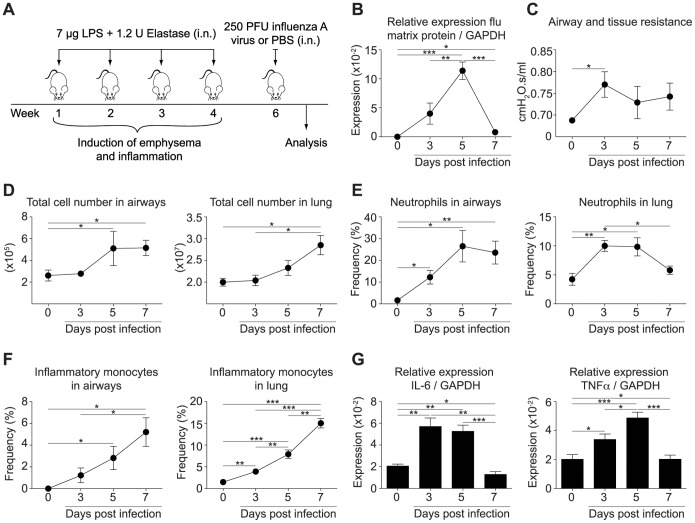
Influenza infection induced exacerbation of established disease in LPS/elastase exposed mice. (A) Experimental protocol of influenza-induced exacerbation of LPS/elastase exposed mice. Control mice (indicated as day 0 in the following) were pre-exposed to LPS/elastase as were their infected counterparts, but challenged only with PBS instead of influenza virus. (B) Viral load of whole lung including airways and trachea was determined by quantitative real-time PCR at the indicated time points post infection or in non-infected mice (day 0) as control, respectively. Expression of influenza matrix protein was normalized to GAPDH. (C) Airway and tissue resistance was assessed by invasive plethysmography. (D) Absolute number of cells in the airways and lung was determined, and (E) the proportion of neutrophils and (F) inflammatory monocytes recruited to airways and lungs upon infection (day 3–7) or PBS challenge (day 0) was analyzed by flow cytometry. (G) Expression of IL-6 and TNFα was assessed by real-time PCR and normalized to GAPDH. (B)–(G) Experiments were performed in BALB/c mice and results are representative of at least two independent experiments (n = 4–5). Error bars represent s.e.m.; i.n. (intranasal).

To study viral-induced exacerbations, mice were infected with influenza virus 2 weeks after the last LPS/elastase challenge, when the acute inflammation caused by LPS/elastase exposure had subsided (Figure 2A). The peak of viral replication was reached 5 days after the infection (Figure 2B) and was followed by a rapid decline in viral titers (Figure 2B) until complete viral clearance at day 9 post infection (data not shown). The efficiency of the influenza infection was strikingly reduced in mice pretreated with LPS/elastase compared to naïve mice, as revealed by significantly lower viral titers at the peak of viral infection, day 5 and day 7 post infection (Figure S2) which was also reflected by reduced amounts of viable virus in the lung on day 5 (data not shown). This is likely to be due to the development of a polyclonal antibody response observed upon LPS/elastase treatment ([37], [38] and data not shown). Thus, the response to the influenza infection was not comparable between LPS/elastase pretreated and naive mice, and was therefore not included in the remainder of the study.

Invasive measurements of pulmonary resistance in LPS/elastase pretreated mice revealed a viral-induced acute exacerbation of airway dysfunction (Figure 2C). Pulmonary resistance can be influenced by a variety of factors, of which the severity of inflammation is likely to play one of the key roles during exacerbations. In line with this we detected a strong inflammatory response upon infection with influenza virus associated with an augmented absolute number of cells infiltrating into the airways and the lung (Figure 2D). The cells were primarily neutrophils (Figure 2E, Figure S3A) and inflammatory monocytes (Figure 2F, Figure S4A). The peak of neutrophilic inflammation was reached at day 5 post infection and neutrophil numbers declined afterwards (Figure 2E, Figure S3A), directly correlating with the kinetics of viral replication (Figure 2B). In line with this we observed increased expression of the proinflammatory cytokines IL-6 and TNFα peaking at day 3 or 5 post infection, respectively, and subsiding at day 7 after the infection (Figure 2G).

Emphysematous damage upon LPS/elastase treatment also resulted in an increase in the lung compliance. However the acute inflammatory changes induced by influenza infection had no impact on the increase in lung compliance (data not shown).

Acute pulmonary dysfunction, neutrophilic inflammation and enhanced levels of proinflammatory cytokines such as IL-6 and TNFα have all been observed during exacerbations of COPD patients, indicating that the viral-induced inflammation in our mouse model is in line with that seen in humans.

### IL-1β Contributes to Lung Dysfunction and Pulmonary Inflammation during Influenza Infection of LPS/elastase Treated Mice

**Figure 3:**
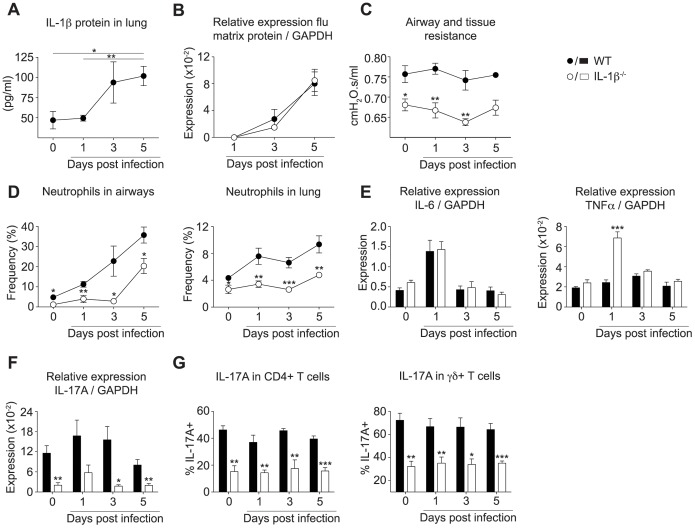
IL-1β mediated airway resistance, neutrophilic inflammation and IL-17A expression during influenza-induced exacerbations of COPD. Exacerbation of COPD in C57BL/6 mice was induced as depicted in Figure 2A. (A) IL-1β protein in whole lung including airways and trachea following influenza infection (day 1–5) or PBS challenge (day 0) was assessed by ELISA. (B) Viral load in whole lung and trachea of wild type or IL-1β deficient animals was determined by quantitative real-time PCR and normalized to GAPDH. (C) Airway and tissue resistance was measured by invasive plethysmography at indicated time points after infection. (D) The proportion of neutrophils in the airways and lung was determined by flow cytometry. Data are pooled from two independent experiments (n = 4–5). (E) Expression of IL-6, TNFα, and (F) IL-17A was assessed by quantitative real-time PCR and normalized to GAPDH. (G) Proportion of IL-17A positive CD4^+^ T cells or γδ T cells was determined by flow cytometry after restimulation *in vitro*. All data are representative of at least two independent experiments (n = 4–5) and mean ± s.e.m. is shown.

IL-1 has previously been shown to be a driving factor in the development of emphysema and inflammation in animal models of COPD [20]–[27]. As we observed an increase of IL-1β protein in the lungs of LPS/elastase treated mice upon influenza infection (Figure 3A), we hypothesized that it also promotes innate immune responses and influences pulmonary function during exacerbations. Consequently, we exposed IL-1β deficient and C57BL/6 wild type mice to LPS/elastase and infected them with influenza virus as described above. The C57BL/6 strain showed similar kinetics of neutrophil and inflammatory monocyte recruitment upon viral exacerbation as observed for the BALB/c mice (Figure 3D, Figure S3B, S4B).

Of note, viral replication was not altered in IL-1β deficient mice (Figure 3B), demonstrating that any effects seen in the absence of IL-1β were not due to differences in the infection rate. C57BL/6 wild type mice exhibited a smaller change in pulmonary resistance in response to viral infection (Figure 3C) in comparison to BALB/c mice (Figure 2C), with a slight increase at day 1 post infection (Figure 3C). Nevertheless, pulmonary resistance in mice lacking IL-1β was significantly reduced already after LPS/elastase exposure alone (Figure 3C), thus supporting the described role for IL-1β in the development of chronic lung disease [20]–[27]. Furthermore, pulmonary resistance in IL-1β-deficient mice was also completely unaffected by the viral challenge (Figure 3C). We did not detect any impact of IL-1β on inflammatory monocytes (Figure S4B); however, we found a decreased frequency and number of neutrophils in non-infected IL-1β deficient mice upon exposure to LPS/elastase (Figure 3D, Figure S3C). Similarly, the recruitment of neutrophils to the airways and lung following viral infection was also significantly abrogated in the absence of IL-1β (Figure 3D, Figure S3C). We observed significantly lower frequencies and absolute numbers of neutrophils including during the peak of neutrophil infiltration and viral replication at day 5 post infection (Figure 3D, Figure S3C). Nevertheless, the control of the virus was unaffected as displayed by unaltered viral titers (Figure 3B).

Considering its strong impact particularly upon neutrophils, we sought to address the mechanisms through which IL-1β mediated this neutrophilic inflammation. Expression of the main neutrophil chemoattractants CXCL1, CXCL2, and CXCL5 were induced upon influenza infection, but were unaffected by IL-1β (Figure S5). In addition we did not detect any influence of IL-1β or the viral infection on the expression of G-CSF (Figure S5). Given there is substantial redundancy in neutrophil chemoattractants we thus next looked upstream at the proinflammatory cytokines IL-17A, IL-6, and TNFα, which can all stimulate neutrophilic inflammation by inducing chemotactic, growth or survival factors [16], [39], [40]. As expression of IL-6 and TNFα were induced in our mouse model upon influenza infection (Figure 2G), we hypothesized that IL-1β drove the observed inflammation by altering their production. The peak expression of IL-6 and TNFα upon influenza infection was reached faster in C57BL/6 wild type mice at day 1 post infection (Figure 2G) as compared to day 3 in BALB/c mice (Figure 3E), and thus coincided with the earlier response in pulmonary resistance observed in the C57BL/6 strain (Figure 3C). IL-6 protein levels followed similar kinetics in both mouse strains (Figure S6A,B), while TNFα protein production was below the sensitivity limit of our assay. However, lack of IL-1β did not impair the expression and production of IL-6 (Figure 3E, Figure S6B) and in fact increased the expression of TNFα (Figure 3E). We therefore focused on IL-17A, a proinflammatory cytokine that has been shown to be elevated in COPD patients [41], [42] and whose induction partially depends on IL-1β [43]–[45]. We found significantly reduced expression levels of IL-17A in lung homogenate in the absence of IL-1β, in both non-infected as well as influenza-infected LPS/elastase exposed mice (Figure 3F). IL-17A production was significantly reduced in the predominant cellular sources of IL-17A, such as the CD4^+^ T cells and the γδ T cells in IL-1β deficient mice (Figure 3G). Further sources of IL-17A such as CD3^+^ CD4^−^ CD8^−^ γδTCR^−^ cells that might comprise NKT cells, and CD3^−^ cells also showed reduced levels of IL-17A in the absence of IL-1β (Figure S7A). However, even in the wild type animals these cells represented a very minor population of cells relative to the IL-17A-producing T cells (Figure S7A). Taken together our data showed that in addition to contributing to lung dysfunction, IL-1β played a key role in driving neutrophilic inflammation during influenza-induced exacerbations, an effect that was tightly linked to IL-17A expression.

### Initial Neutrophil Recruitment during Exacerbations is Mediated by IL-17A can be Abrogated by Treatment with Neutralizing Antibodies

**Figure 4:**
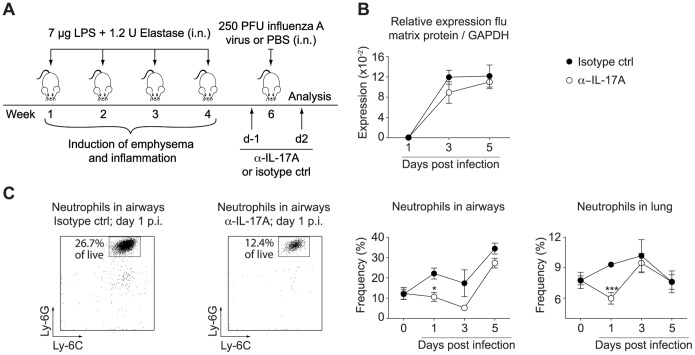
IL-17A mediated neutrophilic inflammation during the initial phase of influenza-induced exacerbation. (A) BALB/c mice were treated with anti-IL-17A (α-IL-17A) or isotype control antibody one day prior and two days after infection with influenza virus (day 1–5 post infection) or PBS challenge (day 0). (B) Viral load was measured by quantitative real-time PCR and normalized to GAPDH. (C) The proportion of neutrophils in airways and lung were assessed by flow cytometry. Two representative FACS plots of airway neutrophilia at day 1 after infection are shown as well as the accumulated data. FACS plots show Ly-6C versus Ly-6G expression of CD11c^−^ CD11b^+^ pre-gated cells and indicate the frequency of neutrophils of all live cells. Data are representative of two independent experiments (n = 4–5), error bars indicate s.e.m; i.n. (intranasal); p.i. (post infection); ctrl (control).

To assess whether IL-17A was indeed a mediator of IL-1β driven neutrophilia we neutralized IL-17A during influenza infection of LPS/elastase treated mice. Neutralization assays were performed in BALB/c mice, which exhibited a similar induction of IL-17A as C57BL/6 mice (Figure S7B). Mice received either an IL-17A neutralizing antibody or an isotype control antibody one day before and two days after the viral infection (Figure 4A). IL-17A neutralization did not impact on the control of viral replication, as viral burden was comparable to the isotype control treated animals (Figure 4B). We found that influenza-induced neutrophil recruitment to the airways and lung was indeed entirely attenuated 24 h after the infection in absence of IL-17A (Figure 4C, Figure S3D). However, neutrophils infiltrated into the lung and airways during the later stages of infection (day 3 and 5 respectively) to finally reach the same frequencies as in mice treated with the isotype control (Figure 4C, Figure S3D); thus indicating that IL-17A was only required for the initial but not for the later recruitment of neutrophils. Hence, IL-1β driven neutrophilia during influenza infection of LPS/elastase exposed mice was mediated by IL-17A in the early phase of infection, but became independent of IL-17A.

### Treatment with Human Recombinant IL-1Ra Impairs Neutrophil Recruitment at the Peak of Viral Replication

**Figure 5:**
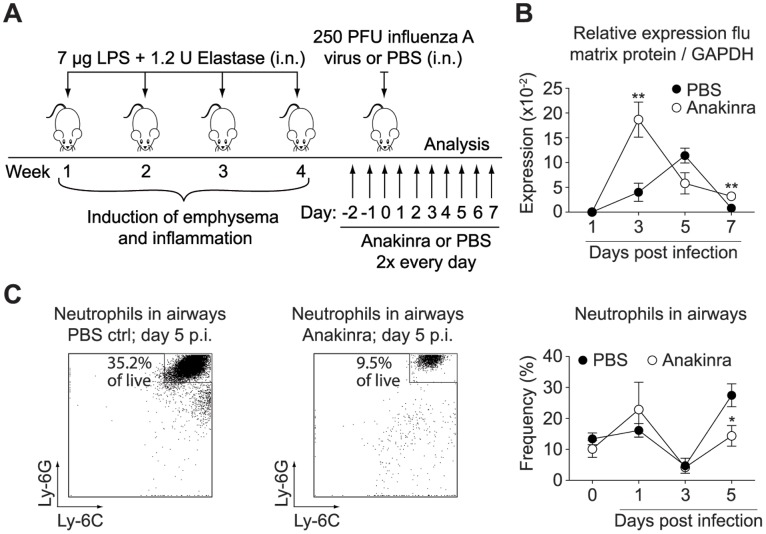
Treatment with Anakinra reduced neutrophil recruitment to the airways at the peak of viral-induced inflammation. (A) BALB/c mice received Anakinra or PBS twice every day, starting two days prior to the viral infection and until mice were sacrificed. (B) Viral load was determined by quantitative real-time PCR and normalized to GAPDH. (C) Proportion of neutrophils recruited to the airways following influenza infection (day 1–5) or PBS challenge (day 0) was assessed by flow cytometry. Two representative FACS plots of each group at day 5 after infection are shown as well as the plotted data. FACS plots show Ly-6C versus Ly-6G expression of CD11c^−^ CD11b^+^ pre-gated cells and indicate the frequency of neutrophils of all live cells. Data are representative of two independent experiments (n = 4–5), error bars indicate s.e.m; i.n. (intranasal); p.i. (post infection); ctrl (control).

Our data showed that a constitutive lack of IL-1β substantially impaired neutrophil infiltration into the airways and lung during influenza-induced exacerbations of chronic lung inflammation. Thus, we sought to assess whether it is sufficient to block IL-1β signaling only during the course of infection, an important determinant regarding a potential therapeutic intervention. Accordingly, recombinant IL-1Ra (Anakinra) or PBS was administered twice daily, starting two days prior to the viral infection (Figure 5A). Mice receiving Anakinra displayed an impaired early control of viral infection leading to a higher viral burden at day 3 post infection, although viral titers rapidly declined afterwards to levels similar to non-treated mice at day 7 post infection (Figure 5B), and virus was completely cleared at day 9 post infection (data not shown). Treatment with Anakinra was efficient in reducing neutrophil frequencies and numbers in the airways at day 5 post infection, the peak of neutrophilic infiltration and viral replication (Figure 5C, Figure S3E). We did not observe an effect of Anakinra on the recruitment of inflammatory monocytes (Figure S4C). Similarly, we did not detect significant changes in lung function upon the treatment with Anakinra (data not shown).

**Figure 6:**
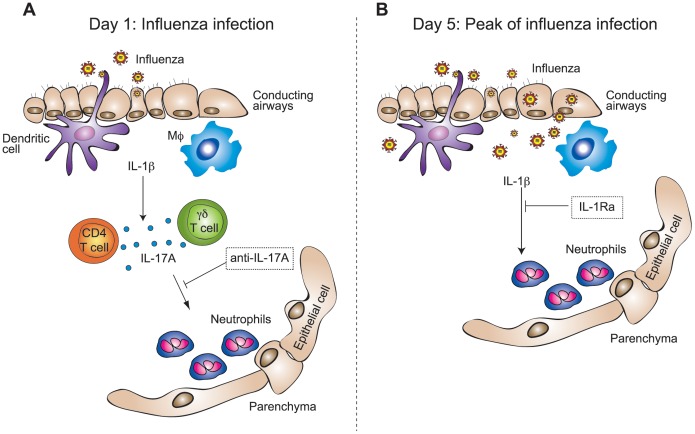
Neutrophilic inflammation during influenza-induced exacerbation of COPD is mediated by IL-1β and IL-17A. (A) In the initial phase of viral replication, at 24 h following the infection, IL-1β-induced IL-17A caused the recruitment of neutrophils to the airways and lung. (B) At the peak of viral replication, day 5 post infection, neutrophilia became independent of IL-17A, but was still mediated by IL-1β. Blocking of IL-17A or IL-1β abrogated neutrophilic inflammation in the early phase of the infection or at the peak of viral replication, respectively.

In conclusion our data demonstrated that IL-1β influenced neutrophilic inflammation during influenza-induced exacerbation of chronic lung inflammation in mice throughout the entire phase of viral replication. Neutrophil recruitment was mediated by IL-17A in the first 24 h following viral challenge and could efficiently be blocked in the early phase of infection by antibodies neutralizing IL-17A. During the peak of inflammation and viral replication, IL-1β driven neutrophilia was independent of IL-17A, but could be significantly reduced by treatment with the IL-1Ra anakinra (Figure 6).

## Discussion

In addition to a constant disease burden, COPD patients suffer from episodes of acute symptom worsening causing a rapid decline in respiratory function that can necessitate hospitalization and even lead to death [46]. Indeed, a meta-analysis study estimated a case-fatality rate of 15.6% following hospitalization due to an exacerbation [47]. As there is a clear need to understand the mechanisms driving exacerbations and responsiveness to therapy, we examined viral-induced exacerbations in mice. In our model (Figure 2A), mice developed a strong inflammatory response characterized by a neutrophilic infiltrate into the airways and lung (Figure 2E), enhanced expression of proinflammatory cytokines such as TNFα and IL-6 (Figure 2G), and impairment in lung function (Figure 2C). Decline in lung function and neutrophil accumulation are characteristic of exacerbations in humans [48]–[50], and elevated levels of TNFα and IL-6 have similarly been measured in the sputum of patients undergoing an exacerbation [17], [51]. Thus, our mouse model reflects key pathological characteristics of COPD exacerbations in humans.

Given the significant differences in susceptibility between the PBS treated and LPS/Elastase treated mice (Figure S2 and data not shown) which might be due to the development of a polyclonal B-cell response upon LPS/Elastase administration, we believe that conclusions can only be drawn from the comparison between stable disease versus episodes of exacerbation. Notably, this is also supported by findings in cigarette smoke (CS) models of pulmonary inflammation where CS exposed mice showed a considerably altered response to influenza infection [37] as well as the accelerated clearance of *Haemophilus influenza* [38] in comparison to control mice.

In this study we specifically focused on the role of the proinflammatory cytokine IL-1β. IL-1 signaling has been shown to be essential for the recruitment of neutrophils in other mouse models mimicking the pathology of stable COPD, such as exposure to cigarette smoke [20], [21], [23], [26] or to elastase alone [22]. Building upon these studies we found that during influenza-induced exacerbations, pulmonary accumulation of neutrophils was also driven by IL-1β (Figure 3D, Figure S3C). Our data is in line with results from a study of Botelho et al. [20] who investigated IL-1 in a mouse model of acute cigarette smoke exposure. They found that interleukin-1 receptor (IL-1R) deficiency led to a reduction of neutrophils following infection with influenza and that this effect was independent of IL-1α. One could therefore speculate that IL-1β may play a critical role in neutrophil recruitment in their model as well.

IL-17A, a cytokine that plays a central role in amplifying inflammatory cascades by inducing a variety of chemokines and cytokines, has also been reported to contribute to the development of emphysema [52], [53] and to the immunopathology following influenza infections [54]. In line with this, we found that IL-17A mediated early inflammation in our model of influenza-induced exacerbations of chronic lung disease (Figure 4C). Our data showed that IL-1β driven neutrophil recruitment during the first 24 h of infection was mediated by lL-17A, while it became independent of IL-17A at later time points during the exacerbation. We found that in the absence of IL-1β the expression of IL-17A was completely abrogated upon LPS/elastase exposure as well as during exacerbations (Figure 3F), thus demonstrating that IL-1β is required for the induction of IL-17A. IL-17A expression was induced by LPS/elastase exposure alone, and levels were maintained throughout the viral-induced exacerbation (Figure 3F,G); however, while IL-1β levels increased during the later phase of infection (Figure 3A), IL-17A expression surprisingly remained unaltered and even decreased at the peak of viral replication (Figure 3F). Nevertheless, we could block the recruitment of neutrophils at early time points following the influenza infection by neutralizing IL-17A (Figure 4C). During the peak of viral replication, and thus a more severe state of inflammation, IL-17A neutralization could not prevent neutrophil influx. This might be due to an induction of cytokines during the progression of the infection, which could overcome the effect of IL-17A. Therefore, treatment with anti-IL-17A seems to be favorable in the early phases of exacerbations while blocking IL-1β might be more advantageous during the ongoing infection. Whether these findings translate into a clinical setting remains to be investigated.

Keeping in mind that neutrophil recruitment is elevated in the vast majority of cases of COPD exacerbations regardless of their etiology [7], and furthermore that the increase of neutrophils in the sputum correlates with exacerbation severity [7], attenuating neutrophilia could be beneficial in patients undergoing a viral-induced exacerbation. Current treatment relies mainly on corticosteroids and bronchodilators that have been shown to reduce the frequency of exacerbations [55]–[57], but have no positive effect on an ongoing episode of exacerbation. Indeed, no reduction in the inflammatory response including neutrophil influx in mice and cytokine expression in humans could be achieved by treatment with steroids during COPD exacerbations [17], [19]. In contrast, corticosteroids have been shown to actually support neutrophil survival [58], [59]. Moreover, treatment with corticosteroids, efficient in reducing IL-1β levels in stable COPD, did not affect the amount of IL-1β protein in exhaled breath condensate during exacerbations [17]. As blocking of either IL-17A (Figure 4C) or IL-1β (Figure 3D, Figure S3C) efficiently reduced neutrophilic inflammation during viral exacerbation, these two molecules could be considered potential targets for therapeutic intervention. Given the redundancies in these two inflammatory pathways, a combination therapy of anti-IL-17A and anti-IL-1β may prove more beneficial in improving lung function.

Within the context of targeting IL-17A or IL-1β in the clinic, one has to keep in mind that altering proinflammatory immune responses harbors the risk of interfering with the control of acute infection and of susceptibility to opportunistic infections. To shorten the duration of intervention and thereby reducing the risk of prolonged or secondary infections, we treated mice directly before and during the viral infection with anti-IL-17A (Figure 4A) or IL-1Ra (Figure 5A). This was sufficient to abrogate neutrophil recruitment at the indicated time points. Furthermore, neutralizing IL-17A did not promote elevated viral replication (Figure 4B). Treatment with IL-1Ra, and thereby blocking both IL-1α and IL-1β, led to increased viral titers in the initial phase of infection (Figure 5B), whereas the absence of IL-1β alone did not affect viral replication (Figure 3B). It is therefore likely that in our model the initial control of the virus might be mediated rather by IL-1α than by IL-1β. This is in line with data from Botelho et al. [20], who found similarly elevated viral titers upon neutralization of IL1-α as in the complete absence of IL-1R. Our data thus suggest that targeting specifically IL-1β, and not its receptor, would be favorable in a therapeutic application. However, as treatment with Anakinra did not interfere with final viral clearance it still could be considered as a therapeutic approach with the additional advantage of already being used in the clinic for other indications.

Taken together our data demonstrated that blocking of IL-17A or IL-1β signaling during influenza-induced exacerbations diminished neutrophilic infiltration at distinct phases of infection. Whether those mechanisms apply also to other respiratory viral infections remains to be elucidated, however is plausible given the common early inflammatory pathways induced by respiratory viral infections. Overall, blockade of IL-17A and IL-1β could be valuable therapeutic options for future treatment of viral induced exacerbations of chronic lung inflammation.

## Supporting Information

Figure S1 Identification of cell subsets in airways and lung by flow cytometry. Cells from lung digest or bronchoalveolar lavage of the airways were characterized by flow cytometry. (A) Neutrophils were distinguished by absence of CD11c, and by high expression of CD11b, Ly-6G, and Ly-6C, while inflammatory monocytes were defined as CD11c^−^ CD11b^+^ Ly-6C^+^ Ly-6G^low−intermediate^. (B) CD4^+^ T cells were characterized by positive staining for CD4 and γδ T cells were distinguished by expression of CD3 and the γδ TCR. Gates were set according to fluorescence minus one controls where applicable.
(TIF)

Figure S2 Impaired infection by influenza virus in mice pretreated with LPS/elastase. Naïve or LPS/elastase treated BALB/c mice were infected with influenza virus as described in the materials and methods. The viral load was determined in whole lung including airways and trachea by quantitative real-time PCR and normalized to GAPDH. Data are representative of three independent experiments (n = 4–5).
(TIF)

Figure S3 Absolute numbers of neutrophils during exacerbations reflect the same results as neutrophil frequencies. Absolute numbers of neutrophils recruited to the airways and lungs upon infection (day 1–7) or PBS challenge (day 0) of LPS/elastase pretreated mice were calculated according to cell frequencies determined by flow cytometry (Figure 1–4) and total cell influx. Absolute numbers of neutrophils are shown for (A) BALB/c wild type mice, (B) C57BL/6 wild type mice, (C) IL-1β deficient mice, (D) mice treated with anti-IL-17A (α-IL-17A) or isotype control antibody, and (E) for mice treated with anakinra or PBS respectively. (D) **^+^**(*p = 0.06)*. (A)–(B), (D)–(E) Results are representative of at least two independent experiments (n = 4–5). (C) Data are pooled from two independent experiments (n = 4–5). Error bars represent s.e.m.
(TIF)

Figure S4 Inflammatory monocytes were induced during the exacerbation but unaffected by IL-1β deficiency. Total numbers of inflammatory monocytes recruited to airways and lungs were calculated according to cell frequencies determined by flow cytometry and total cell influx. Inflammatory monocyte influx is shown for (A) BALB/c wild type mice, (B) IL-1β deficient mice, and (C) for mice treated with anakinra or PBS, respectively. (A)–(C) Results are representative of at least two independent experiments (n = 4–5). Error bars represent standard error of the mean (s.e.m.).
(TIF)

Figure S5 IL-1β did not influence the expression of the neutrophil chemoattractants CXCL1, CXCL2, CXCL5, or G-CSF. Expression of CXCL1, CXCL2, CXCL5, and G-CSF in whole lung including airways and trachea of C57BL/6 wild type and IL-1β deficient mice was assessed by quantitative real-time PCR and normalized to GAPDH. For CXCL1, CXCL2 and CXCL5 data are pooled from two independent experiments (n = 4–5), for G-CSF data are representative of two independent experiments (n = 4–5). Error bars indicate s.e.m. Filled circles indicate wild type, open circles IL-1β deficient mice.
(TIF)

Figure S6 IL-6 protein followed similar kinetics in C57BL/6 and BALB/c mice and was unaffected by IL-1β. IL-6 protein in lung homogenate following viral infection of LPS/elastase exposed mice was determined by ELISA in (A) BALB/c and (B) C57BL/6 wild type mice as well as in (B) IL-1β deficient mice. Results are representative of (A) two and (B) three independent experiments (n = 3–5). Error bars indicate s.e.m.
(TIF)

Figure S7 Induction of IL-17A in BALB/c mice and additional sources of IL-17A during exacerbations. (A) Proportion of IL-17A positive CD3^+^ CD4^−^ CD8^−^ γδTCR^−^ cells or CD3^−^ cells was determined by flow cytometry after unspecific restimulation *in vitro*. (B) IL-17A expression in BALB/c mice was assessed in lung homogenate by quantitative real-time PCR and normalized to GAPDH. All data are representative of two independent experiments (n = 4–5) and mean ± s.e.m. is shown.
(TIF)
