# Supplementary figures and images for: Targeting IL-1β and IL-17A Driven Inflammation during Influenza-Induced Exacerbations of Chronic Lung Inflammation
Source: PLoS One. 2014 Jun 11;9(6):e98440. doi: 10.1371/journal.pone.0098440 (PMC4053370; doi:10.1371/journal.pone.0098440)

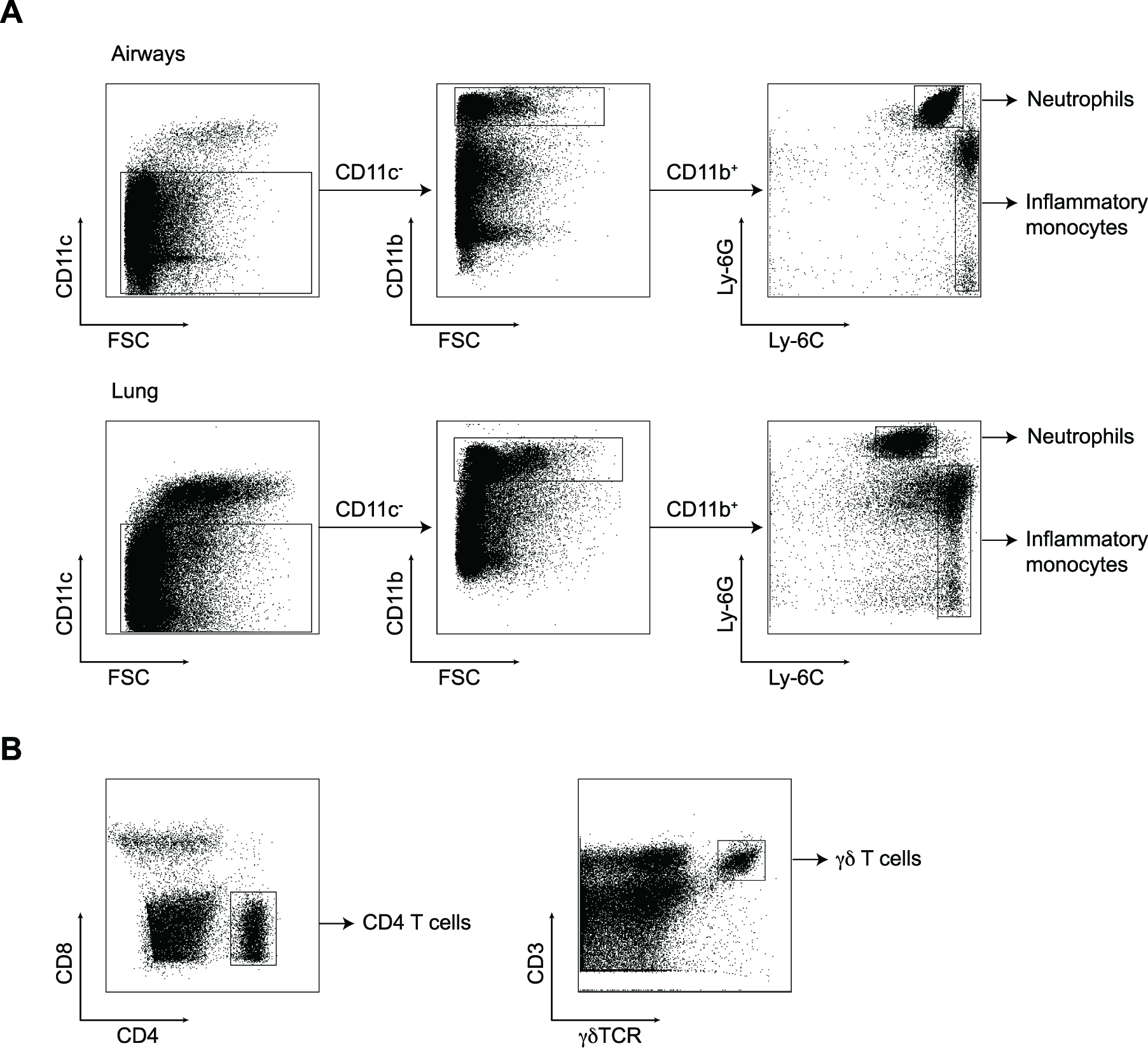

Supplement: Figure S1 — Identification of cell subsets in airways and lung by flow cytometry. Cells from lung digest or bronchoalveolar lavage of the airways were characterized by flow cytometry. (A) Neutrophils were distinguished by absence of CD11c, and by high expression of CD11b, Ly-6G, and Ly-6C, while inflammatory monocytes were defined as CD11c− CD11b+ Ly-6C+ Ly-6Glow−intermediate. (B) CD4+ T cells were characterized by positive staining for CD4 and γδ T cells were distinguished by expression of CD3 and the γδ TCR. Gates were set according to fluorescence minus one controls where applicable. (TIF) [file pone.0098440.s001.tif]

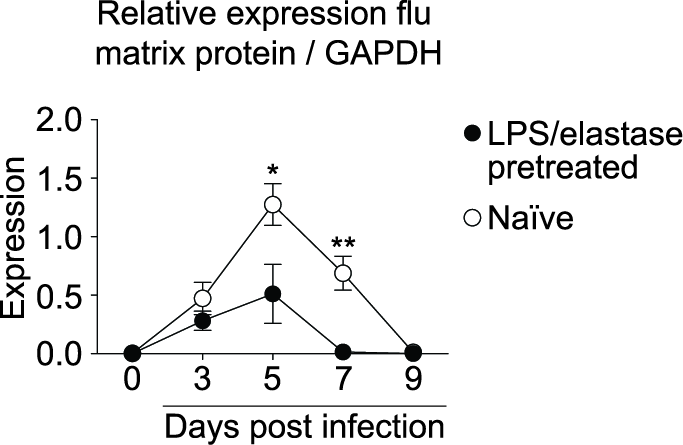

Supplement: Figure S2 — Impaired infection by influenza virus in mice pretreated with LPS/elastase. Naïve or LPS/elastase treated BALB/c mice were infected with influenza virus as described in the materials and methods. The viral load was determined in whole lung including airways and trachea by quantitative real-time PCR and normalized to GAPDH. Data are representative of three independent experiments (n = 4–5). (TIF) [file pone.0098440.s002.tif]

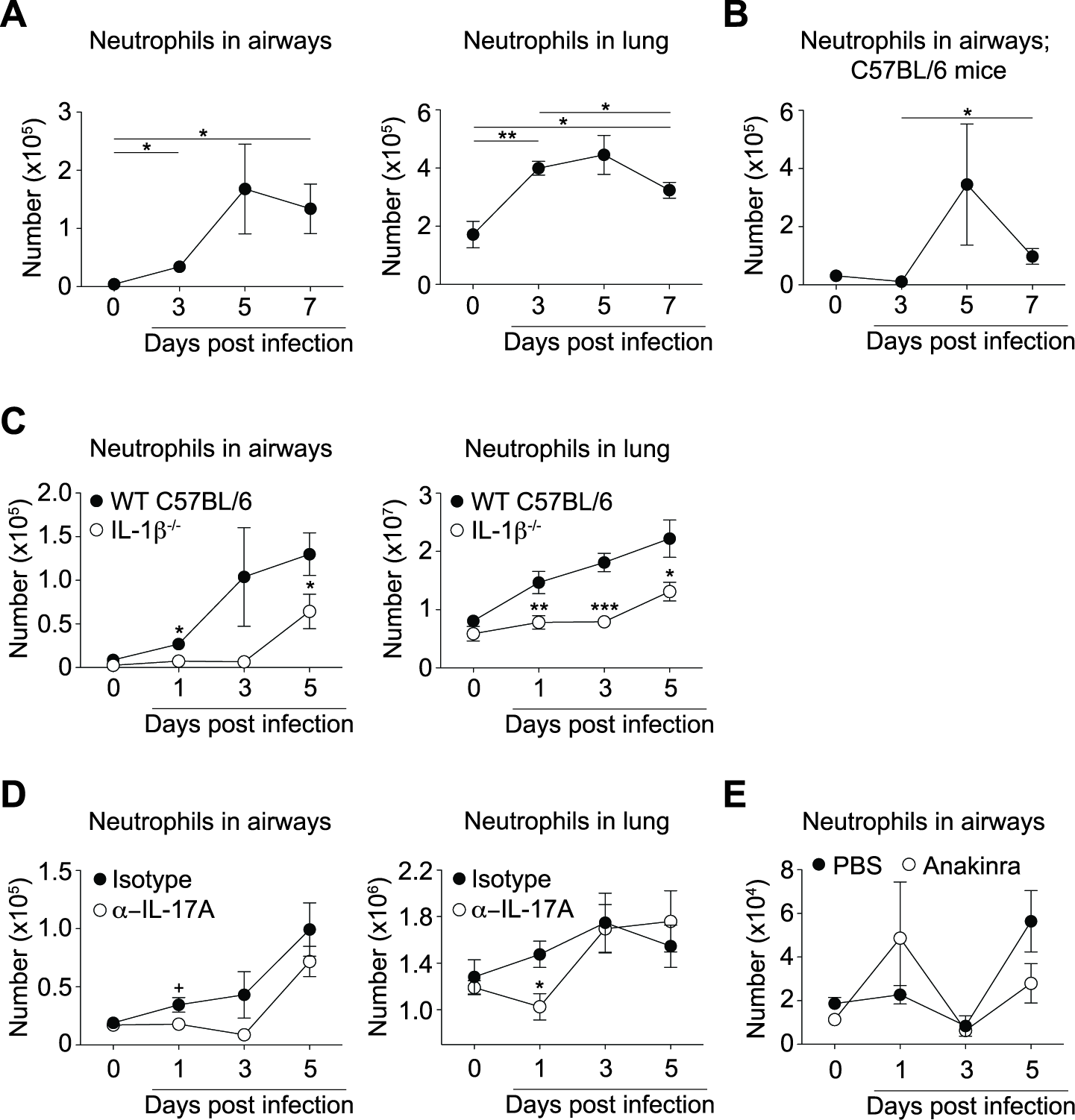

Supplement: Figure S3 — Absolute numbers of neutrophils during exacerbations reflect the same results as neutrophil frequencies. Absolute numbers of neutrophils recruited to the airways and lungs upon infection (day 1–7) or PBS challenge (day 0) of LPS/elastase pretreated mice were calculated according to cell frequencies determined by flow cytometry (Figure 1–4) and total cell influx. Absolute numbers of neutrophils are shown for (A) BALB/c wild type mice, (B) C57BL/6 wild type mice, (C) IL-1β deficient mice, (D) mice treated with anti-IL-17A (α-IL-17A) or isotype control antibody, and (E) for mice treated with anakinra or PBS respectively. (D) +(p = 0.06). (A)–(B), (D)–(E) Results are representative of at least two independent experiments (n = 4–5). (C) Data are pooled from two independent experiments (n = 4–5). Error bars represent s.e.m. (TIF) [file pone.0098440.s003.tif]

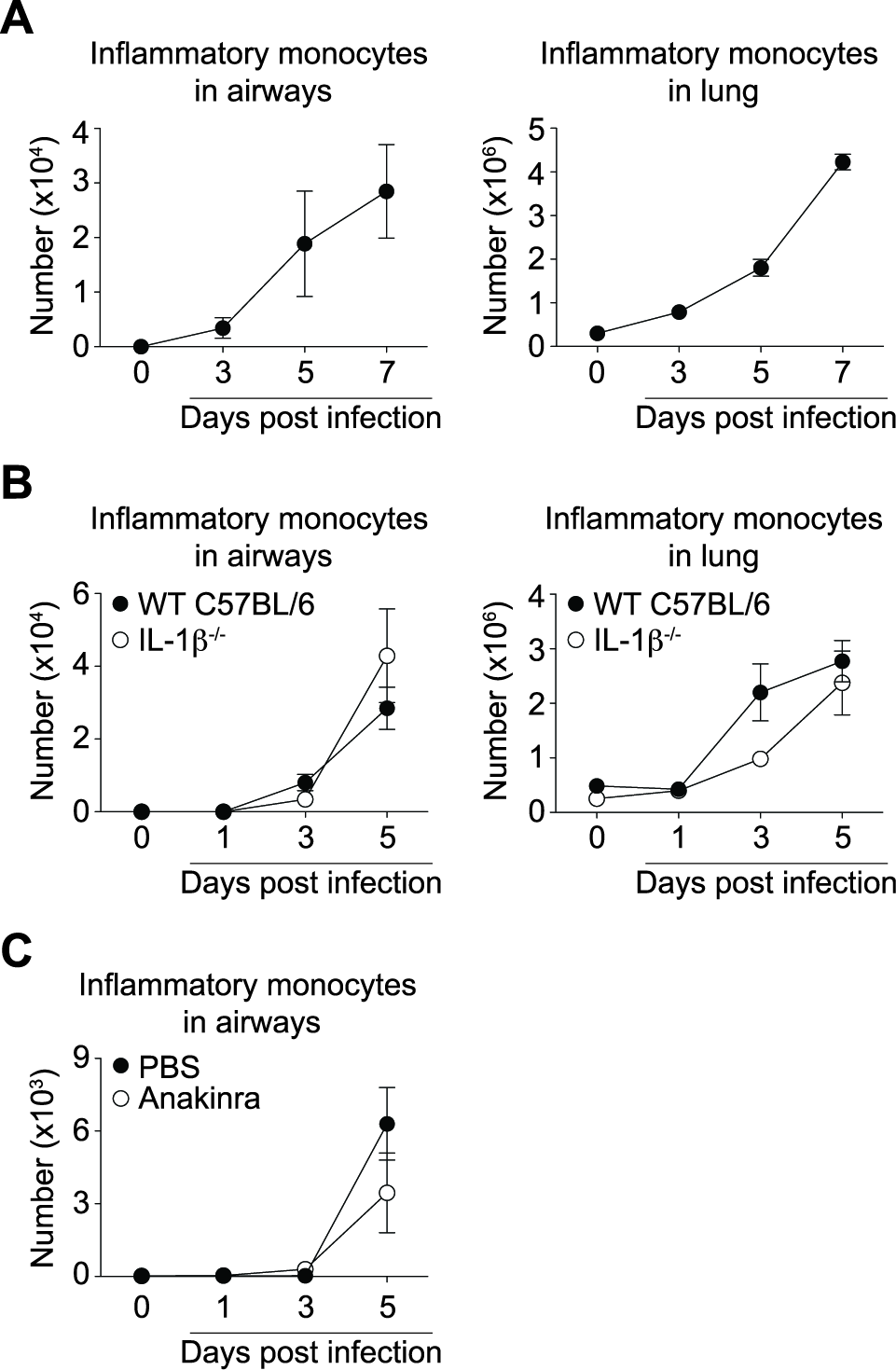

Supplement: Figure S4 — Inflammatory monocytes were induced during the exacerbation but unaffected by IL-1β deficiency. Total numbers of inflammatory monocytes recruited to airways and lungs were calculated according to cell frequencies determined by flow cytometry and total cell influx. Inflammatory monocyte influx is shown for (A) BALB/c wild type mice, (B) IL-1β deficient mice, and (C) for mice treated with anakinra or PBS, respectively. (A)–(C) Results are representative of at least two independent experiments (n = 4–5). Error bars represent standard error of the mean (s.e.m.). (TIF) [file pone.0098440.s004.tif]

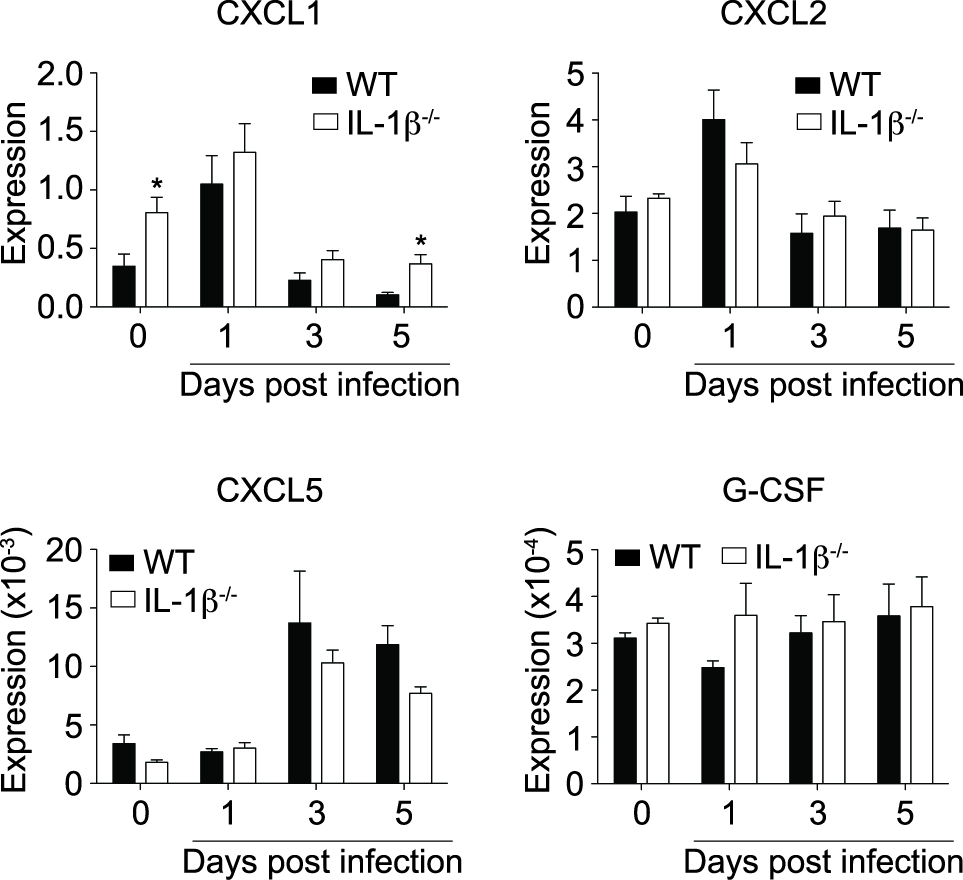

Supplement: Figure S5 — IL-1β did not influence the expression of the neutrophil chemoattractants CXCL1, CXCL2, CXCL5, or G-CSF. Expression of CXCL1, CXCL2, CXCL5, and G-CSF in whole lung including airways and trachea of C57BL/6 wild type and IL-1β deficient mice was assessed by quantitative real-time PCR and normalized to GAPDH. For CXCL1, CXCL2 and CXCL5 data are pooled from two independent experiments (n = 4–5), for G-CSF data are representative of two independent experiments (n = 4–5). Error bars indicate s.e.m. Filled circles indicate wild type, open circles IL-1β deficient mice. (TIF) [file pone.0098440.s005.tif]

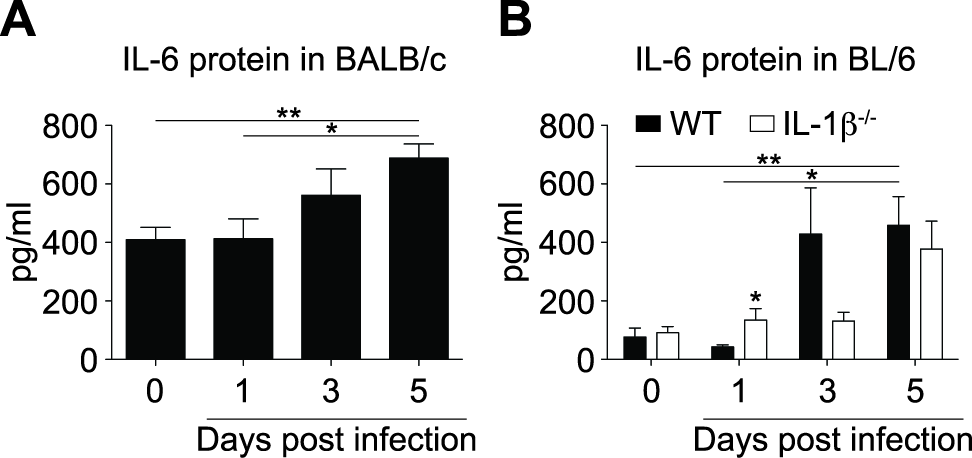

Supplement: Figure S6 — IL-6 protein followed similar kinetics in C57BL/6 and BALB/c mice and was unaffected by IL-1β. IL-6 protein in lung homogenate following viral infection of LPS/elastase exposed mice was determined by ELISA in (A) BALB/c and (B) C57BL/6 wild type mice as well as in (B) IL-1β deficient mice. Results are representative of (A) two and (B) three independent experiments (n = 3–5). Error bars indicate s.e.m. (TIF) [file pone.0098440.s006.tif]

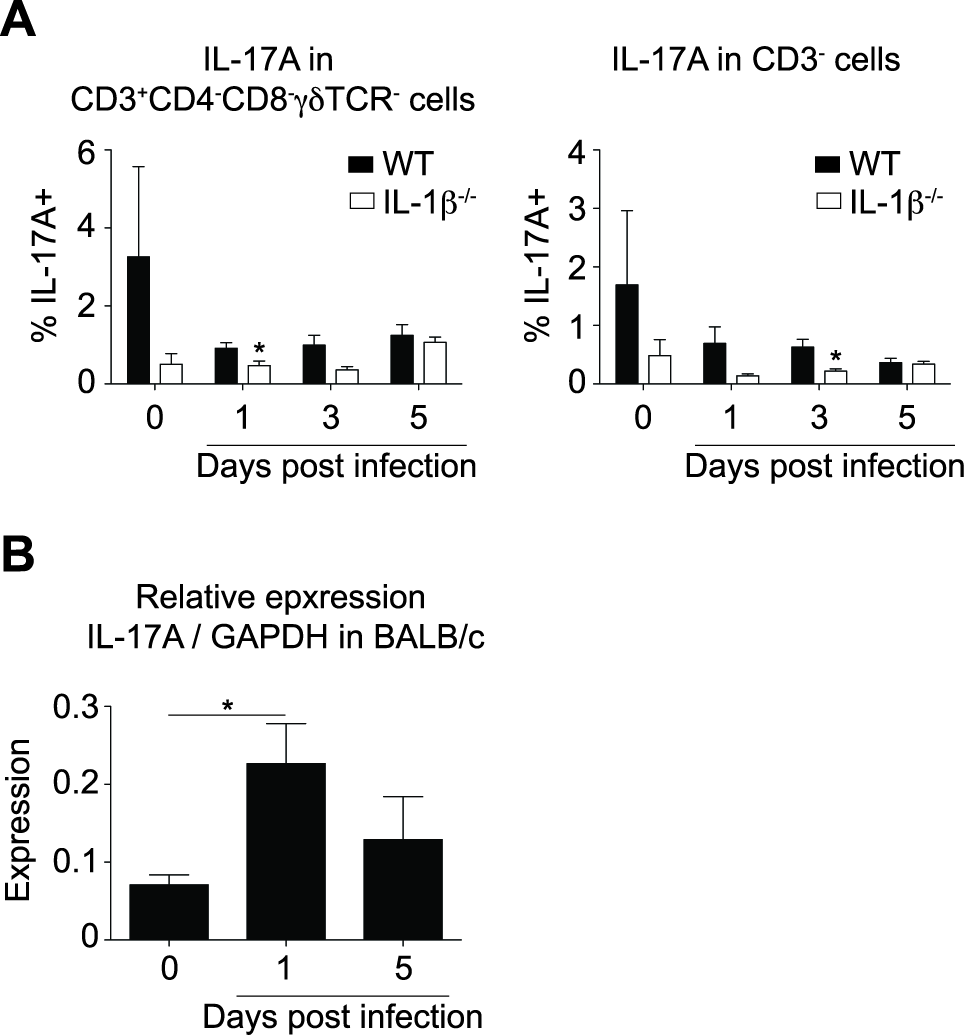

Supplement: Figure S7 — Induction of IL-17A in BALB/c mice and additional sources of IL-17A during exacerbations. (A) Proportion of IL-17A positive CD3+ CD4− CD8− γδTCR− cells or CD3− cells was determined by flow cytometry after unspecific restimulation in vitro. (B) IL-17A expression in BALB/c mice was assessed in lung homogenate by quantitative real-time PCR and normalized to GAPDH. All data are representative of two independent experiments (n = 4–5) and mean ± s.e.m. is shown. (TIF) [file pone.0098440.s007.tif]
